# Compensation Method for Sensor Network Clock Error Based on Cyclic Symmetry Algorithm

**DOI:** 10.3390/s20061738

**Published:** 2020-03-20

**Authors:** Hailiang Feng, Zhanxin Yang, Yuhai Shi, Narjes Nabipour

**Affiliations:** 1 School of Information and Communication Engineering, Communication University of China, Beijing 100024, China; yangzx@cuc.edu.cn; 2 Internet Technology Institute, Academy of Broadcasting Science, Beijing 100866, China; shiyuhai@abs.ac.cn; 3 Institute of Research and Development, Duy Tan University, Da Nang 550000, Vietnam

**Keywords:** cyclic symmetry algorithm, sensor, network, clock, error, compensation method

## Abstract

Since the existing methods cannot evaluate the time delay of different layers of sensor networks, there are some problems such as the low precision of clock error compensation, high time delay, and low efficiency of communication in sensor networks. To solve this problem, a method of clock error compensation in sensor networks based on a cyclic symmetry algorithm is proposed. Based on the basic theory of cyclic symmetry, the cyclic symmetry matrix of the sensor network is constructed; in the communication process, all nodes are extended to get the cumulative delay rate of the sensor network in the specified time domain. Using the cumulative delay rate of the cyclic network and the sensor network, the autoregressive integral sliding mode control model is established to compensate for the cumulative error of clock synchronization. The simulation results show that the compensation accuracy of this method is more than 98%, which can effectively reduce the delay of sensor network. It improves the communication efficiency of the sensor network, meets the communication requirements of the sensor network, and has a very broad application prospect.

## 1. Introduction

Due to the shrinking size of hardware devices and the rapid development of software computing speed, wireless sensor networks are put into use. Wireless sensor networks are powerful and versatile [1]. At present, wireless sensor network applications cover many fields such as public safety, security intensification, health monitoring, intelligent transportation, environmental monitoring, battlefield reconnaissance, target tracking, and emergency location and navigation of fire scenes [2].

Wireless sensor networks can implement functions such as area detection, information collection, distributed processing, information fusion, and data transmission, which can replace human beings in the face of dangerous environments or environmental monitoring that humans cannot reach [3]. In the military field, wireless sensor networks can be used to monitor conflict zones, reconnaissance local terrain and arming, and detect nuclear, biological, and chemical attacks or hazardous materials. It can also monitor enemy movements, monitor enemy forces and equipment, locate and track targets, and provide a basis for decision-making [4]. In the field of industrial control, wireless sensor networks can realize industrial control process monitoring, industrial waste gas monitoring, and promote the intelligent production equipment and flexible production methods [5]. In the agricultural field, wireless sensor networks collect and control environmental information, animal and plant information monitoring, agricultural irrigation automation control technology, grassland humidity, and animal feeding [6]. In the field of smart grids, wireless sensor networks store and correlate electricity information, provide power-saving feedback services, form an efficient and environmentally-friendly smart grid system, integrate power flow, information flow, and business flow, and improve grid operation capability. In the medical field, wireless sensor networks are used in drug management, surveillance monitoring, telemedicine, etc. [7]. The next step will be to integrate the medical system, achieve resource sharing, and ultimately establish a coordinated, collaborative medical system that provides personalized health services.

At present, wireless sensor networks have achieved fruitful results in basic theories and key technologies such as network protocols, wireless communications, data management, network security, and clock synchronization. However, wireless sensor networks still have shortcomings such as limited power, limited computing power, and limited storage capacity. The battery energy of the sensor node is limited. On the one hand, due to the complex environment of the node deployment area and the wide distribution area, many areas are even unreachable, so energy is difficult to supplement. On the other hand, due to the size and cost of the node, it is not possible to use a large-capacity battery or a solar battery. The limited energy of the module has seriously affected the widespread use of wireless sensor networks [8]. How to make the node consume the least amount of energy during use and maximize the service life of the network becomes the main problem for the development of wireless sensor networks. The cost of the sensing node is low, the volume is small, and the carrying capacity is small. These limitations inevitably result in a weak processor capability of the CPU and a small memory capacity. Sensor nodes cover a large area, and the communication range is severely limited by the application environment. The energy consumption of wireless communication increases exponentially with increasing communication distance [9]. However, the node’s wireless communication loan is limited—usually only a few hundred Kbps—so in order to avoid communication terminals, data transmission uses a multi-hop routing mechanism.

Nodes in wireless sensor networks are deployed over a large geographic area, and typically the environment is harsh, causing node failure. Alternatively, it is distributed in a small range with high density, large number, nodes reaching tens of thousands, and even adding new nodes to meet monitoring and other requirements [10]. Therefore, wireless sensor networks are required to be highly robust to meet application requirements. As can be seen from the above challenges, the energy consumption of nodes is the most important part of extending the life cycle of the network. The compensation of the sensor network clock error can effectively improve the life cycle of the wireless sensor network.

In order to improve the survivability of wireless sensor nodes, realize the high robustness of wireless sensor network, prolong the network life cycle, achieve high clock error compensation accuracy, low communication delay, high communication efficiency, and other goals, a clock error compensation method based on the cyclic symmetry algorithm is proposed. This method provides a new idea for the further development of sensor network-related technology, improves the clock error compensation precision and communication efficiency, and is a great progress of modern communication technology, so the research method has a very broad application prospect.

## 2. Materials and Methods

### 2.1. Basic Theory of Cyclic Symmetry

A symmetrical structure means that the geometry of a structure or physical model is regularly arranged by several identical areas. The dynamic properties of the entire structure can be achieved by analyzing a regional structure [11]. Common symmetry forms are axisymmetric, reflective, and cyclically symmetric. For a sensor network, if the structure is rotated at an angle around the axis and the structure (including the material constant) is exactly the same as before the rotation, this structure is called the cyclic symmetrical structure. The minimum angle of rotation that meets this condition is called the cycle period. N=2π/α is an integer called the cyclic symmetry order. The entire structure can be divided into N identical sectors around the axis, each sector differing by α angles. Depending on the cyclic symmetry, the equation of motion of the entire sensor system can be reduced to a basic intra-sector analysis [12]. The basic sector has a degree of freedom of n. The eigenvalue problem of the nN-order cyclic symmetry structure can be transformed into the eigenvalue problem of the order of 1/N orders of the original N/2 with n different pitches.

The theory of cyclic symmetry consists of an infinitely long chain of structures consisting of a series of equivalent substructures [13]. When the free wave propagates in it, the complex propagation constant μ can be used to describe the propagation behavior of the free wave in the structure. The fluctuation amplitude ratio of the adjacent substructure corresponds to $e^{\mu }$. If μ is a pure imaginary number, the corresponding vibration constraint can be obtained after the constraint is added, and the constraint becomes a complex constraint.

Assume that the d sector of an N-order cyclic symmetry has a cyclic symmetric interface of $t_{k}$ and $t_{k}^{\prime }$ with $t_{k}^{\prime }=t_{k}+1$. A local coordinate system is established for each sector of the cyclic symmetry structure, and the local coordinate system also has the characteristics of cyclic symmetry. The node displacement vector of sector $S_{k}$ in its local coordinate system is written as:

(1) $$\delta_{k}=\left\{\begin{array}{c} \delta_{kt}\\ \delta_{kg} \end{array}\right\},\left(k=0,1,2,\cdots ,N-1\right)$$

where $\delta_{kt}$ denotes a node displacement vector on the cyclic symmetry interface $t_{k}$, and $\delta_{kg}$ denotes a node displacement vector inside the sector $S_{k}$. The node displacement vector of the extended sector $S_{k}$ is $-\delta_{k}=\left\{\begin{array}{c} \delta_{kt}\\ \delta_{kg}\\ \delta_{kt}^{\prime } \end{array}\right\}$, and $\delta_{kt}^{\prime }$ represents the node displacement vector on the cyclic symmetric interface, which is obviously known as $\delta_{kt}^{\prime }=\delta_{\left(k+1\right)t}$.

The displacement vector of the overall structure of the sensor is recorded as:

(2) $$\delta =\left\{\begin{array}{c} \delta_{0}\\ \delta_{1}\\ \vdots \\ \delta_{N-1} \end{array}\right\}.$$

From the cyclic symmetry of the structure, the mass and stiffness matrix of the extended sector in its local coordinate system are as follows (k=1,2,⋯,N).

(3) $$m^{1}=m^{2}=\cdots =m^{N}=\left[\begin{array}{ccc} m_{tt} & m_{tg} & m_{tt^{\prime }}\\ m_{gt} & m_{gg} & m_{gt^{\prime }}\\ m_{t^{\prime }t} & m_{t^{\prime }g} & m_{t^{\prime }t^{\prime }} \end{array}\right]$$

(4) $$k^{1}=k^{2}=\cdots =k^{N}=\left[\begin{array}{ccc} k_{tt} & k_{tg} & k_{tt^{\prime }}\\ k_{gt} & k_{gg} & k_{gt^{\prime }}\\ k_{t^{\prime }t} & k_{t^{\prime }g} & k_{t^{\prime }t^{\prime }} \end{array}\right]$$

By assembling the mass stiffness matrix of each sector in the sensor [14], the mass matrix of the overall sensor structure can be obtained (M and K).

Set $K_{1}=\left[\begin{array}{cc} k_{t^{\prime }t^{\prime }}+k_{tt} & k_{tg}\\ k_{g^{\prime }} & k_{gg} \end{array}\right]$, $K_{2}=\left[\begin{array}{cc} k_{tt^{\prime }} & 0\\ k_{gt^{\prime }} & 0 \end{array}\right]$.

Then,

(5) $$K=\left[\begin{array}{cccccc} K_{1} & K_{2} & 0 & \cdots & 0 & K_{2}^{T}\\ K_{2}^{T} & K_{1} & K_{2} & \cdots & 0 & 0\\ \vdots & \vdots & \vdots & \cdots & \vdots & \vdots \\ \vdots & \vdots & \vdots & \cdots & \vdots & \vdots \\ K_{2}^{T} & 0 & 0 & \cdots & K_{2} & K_{1} \end{array}\right].$$

K is a real symmetric block cyclic matrix, and M has a similar form.

The dynamic equation for the overall sensor network without damping is:

(6) $$\dot{M}\delta +K\delta =0$$

If $\xi_{k}=e^{i\left(k-1\right)\alpha }\left(i=\sqrt{-1},k=1,2,\cdots ,N\right)$, the sensor network cyclic symmetry matrix is as follows:

(7) $$V=\left[\begin{array}{cccc} V_{0} & V_{1} & \cdots & V_{N-1} \end{array}\right]$$

and

(8) $$V_{jn\times N,n}=\frac{1}{\sqrt{N}}\left\{\begin{array}{c} I_{n}\xi_{1}\\ I_{n}\xi_{2}\\ \vdots \\ I_{n}\xi_{N} \end{array}\right\}$$

where j=0,1,2,⋯,N−1, $I_{n}$ is a n-order unit matrix, which is transformed δ=Vq, and the Formula (8) is multiplied by $V^{H}$ to obtain:

(9) $$\dot{A}\dot{q}+Bq=0.$$

A and B are block diagonal real symmetric matrices, $A=diag\left(\alpha_{r}\right)$, $B=diag\left(\beta_{r}\right)$. Here, r=0,1,2,⋯,N−1. At this point, the dynamic equations of the cyclic symmetry structure are reduced to nN-order characteristic equations:

(10) $$\alpha_{r}{\ddot{q}}_{r}+\beta_{r}q_{r}=0,\;r=0,1,2,\cdots ,N_{f}.$$

The nN-order symmetric positive definite sparse matrix with a bandwidth of p is p≤n, and the calculation of cholesky decomposition is about $O\left(nNp^{2}/2-p^{3}/3+\frac{2}{3}\left(nNp-p^{2}\right)\right)$. The calculation of the Cholesky decomposition of the nth order symmetric positive definite matrix is about $O\left(n^{3}/6\right)$. The calculation of the nN-order matrix is much smaller than the calculation of an nN-order matrix. It can be seen that the cyclic symmetry algorithm can greatly reduce the computational complexity.

When N is even, $N_{f}=N/2$; when N is odd, $N_{f}=\left(N-1\right)/2$.

For each $r\left(r=1,2,\cdots ,N_{f}\right)$, the characteristic equation must have n double roots.

That is, the vibration mode of the sensor network has two orthogonal modes [15].

### 2.2. Compensation Method for Sensor Network Clock Error 

Using traditional methods to compensate the clock error of the sensor network, it is difficult to quantify the delay time of different levels, the anti-delay effect is not ideal, and the communication efficiency of the sensor network is reduced [16]. Based on the above theory of cyclic symmetry, the compensation method for a sensor network clock error is studied.

#### 2.2.1. Cumulative Delay Rate

**Figure 1:**
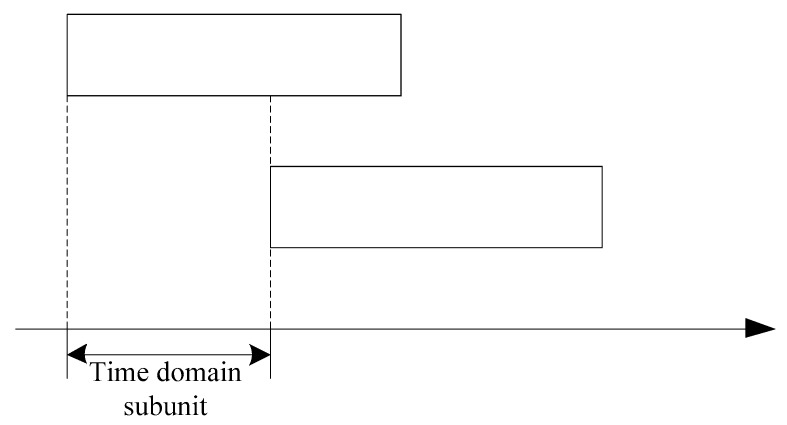
Time domain window structure.

In the process of sensor network clock synchronization cumulative error compensation, the time domain window needs to be divided into several subunits, and each subunit is called a time domain subwindow. In the specified time domain, the clock error approaches a stable value and can be approximated by the m-th derivative. The structure of the time domain is shown in Figure 1.

In the sensor network, the link is randomly selected, and the link is described by g. The time domain corresponding to this link is described by y [17], and the derivation function of the communication signal transmission probability in this window is described by $\chi_{g,y}\left(v\right)$. To make $i_{g,y}\left(v\right)=\mathrm{lg}\chi_{g,y}(v)$, you can use the following formula to expand the nodes in the time domain:

(11) $$i_{g,y}\left(v\right)=\sum_{q!}^{\begin{array}{c} i\left(q\right)\\ g,y \end{array}\left(vp\right)}{\left(v-vp\right)}^{q}+Q\left[{\left(v-vp\right)}^{m}\right]$$

where $\begin{array}{c} i\left(q\right)\\ g,y \end{array}\left(vp\right)/q!$ tends to be stable in the time domain subwindow. Assuming that the requirements of equation $\;c_{g,y,q}=i_{g,y}^{\left(q\right)}\left(vp\right)/q!$ are met, the following formula is obtained for the above formula transformation process:

(12) $$\mathrm{lg}\chi_{g,y}\left(v\right)=\sum_{q=1}^{m}\varphi_{g,y,q}{\left(v-v_{p}\right)}^{q}.$$

The midpoint of the set time domain subwindow is described by $v_{p}$, and the initial moment of the sensor communication signal transmission is described by $v_{0}$. The delay rate of clock synchronization in the sensor network can be calculated using the following formula:

(13) $$\mathrm{lg}r_{k,t}\left(v_{0}\right)=\sum_{q=1}^{M}\mathrm{lg}\chi_{q,y}\left(v_{0}\right).$$

According to the method described above, the sensor network is divided into several subunits, and all nodes are expanded in a specified time domain window [18] to calculate the cumulative delay rate of the sensor network. Communication is the transmission of information. It refers to the transmission and exchange of information from one place to another. The purpose is to transmit messages. The compensation mechanism for clock error of the sensor network can be divided into length compensation and time compensation.

#### 2.2.2. Implementation of Compensation

In order to reduce the delay in the sensor network, an autoregressive integral sliding control model needs to be established, which is described by the following formula:

(14) $$B\left(a^{-1}\right)z\left(l\right)=C\left(a^{-1}\right)v\left(l-1\right)+V\left(a^{-1}\right)\psi \left(l\right)/\Delta$$

where B is the regression control function; C is the link transfer function in the sensor network; ψ is the compensation coefficient in the sensor network; V is the sensor network cyclic symmetry matrix.

In the process of sensor network clock synchronization cumulative error compensation, the objective function can be described by the following formula:

(15) $$K={\sum_{k=1}^{p}\left[z(l+k)-x(l+k)\right]}^{2}+\sum_{k=1}^{n}\nu (k)[\Delta v{\left(l+k-1\right]}^{2}.$$

In the above formula, p is used to describe the number of links in the sensor network, n is used to describe the number of communication signals, v is used to describe the corresponding weight, and z(l+k) is used to describe the clock synchronization error of the k-th link at time l.

In the objective function, the purpose of the last item is to suppress noise interference during communication [19] and improve the accuracy of communication.

For the above objective function recursion [20], the control model that can get the minimum error is as follows:

(16) $$Z^{\prime }=Z_{n}+H\Delta V$$

where,

(17) $$Z^{\prime }={\left[z^{*}(l+1|l),z^{*}(l+2|l),\cdots ,z^{*}(l+p|l)\right]}^{T}$$

(18) $$Z_{n}={\left[z_{n}(l+1),z_{n}(l+2),\cdots ,z_{n}(l+p)\right]}^{T}$$

(19) $$\Delta V={\left[\Delta v\left(l\right),\Delta v\left(l+1\right),\cdots ,\Delta v\left(l+p+1\right)\right]}^{T}$$

(20) Δv(l+j)=v(l+j)−v(l+j−1)

(21) $$H=\left[\begin{array}{cccc} c_{1,0} & 0 & \cdots & 0\\ c_{2,0} & c_{1,0} & \cdots & 0\\ \vdots & \vdots & \vdots & \vdots \\ c_{p,0} & c_{p-1,0} & \cdots & c_{1,0} \end{array}\right].$$

The vector form of the objective function of the sensor network clock synchronization cumulative error compensation is described by the following formula:

(22) $$K={\left(Z-X\right)}^{T}\left(Z-X\right)+v\Delta V^{T}\Delta V.$$

The compensation of the clock synchronization cumulative error of the sensor network is realized according to the following formula:

(23) $$v\left(l\right)=v\left(l-1\right)+\Delta v\left(l\right)=v\left(l-1\right)+\left[1,0,\cdots ,0\right]{\left(H^{T}H+\mu J\right)}^{-1}H^{T}\left[X-Z_{n}\right].$$

According to the above method, an autoregressive integral sliding control model is established, and the clock synchronization cumulative error compensation of the sensor network is realized.

## 3. Results

In order to verify the effectiveness of this method in compensating for the clock error of sensor networks, a simulation experiment is carried out. The overall experimental scheme is to set up the simulation experiment environment, the operating system is Windows 10, and the simulation software is Matlab 7.0. Experimental sample data of a large sensor network application provider in this city will take the part of the background data as the experimental sample data after negotiation. The CRLB method and clock synchronization accumulation method were selected as experimental comparison methods, and the communication delay rate, communication efficiency, information overhead, error detection accuracy, compensation accuracy, computational complexity, and clock drift of unknown nodes were selected as experimental comparison indexes. The influencing factors of clock error compensation in sensor networks are noise interference during information transmission and subjective factors in the process of experimental data acquisition. The experimental environment settings are shown in Table 1.

**Figure 2:**
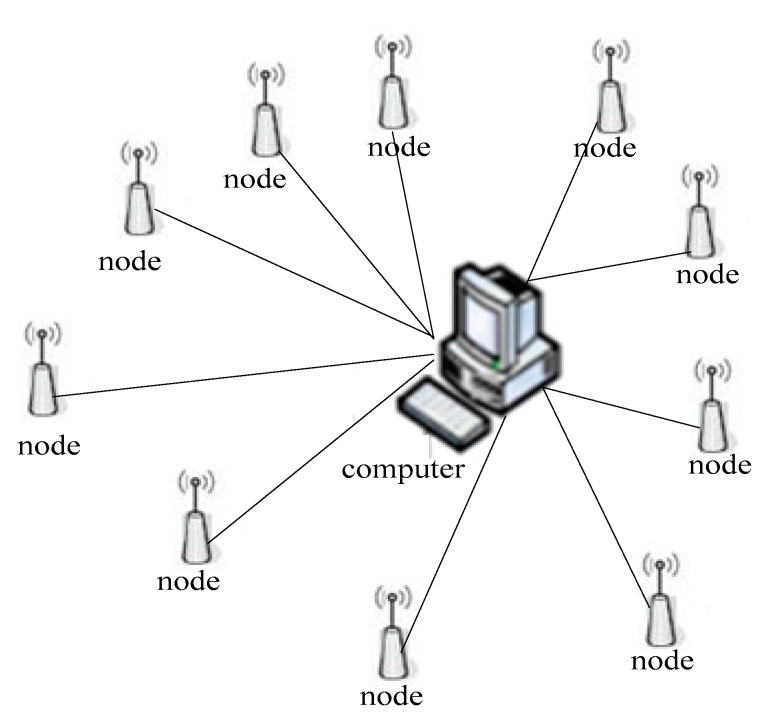
Sensor communication network node distribution map.

Set the number of sensing communication signals to 100, and the distribution of sensor network nodes is shown in Figure 2.

**Figure 3:**
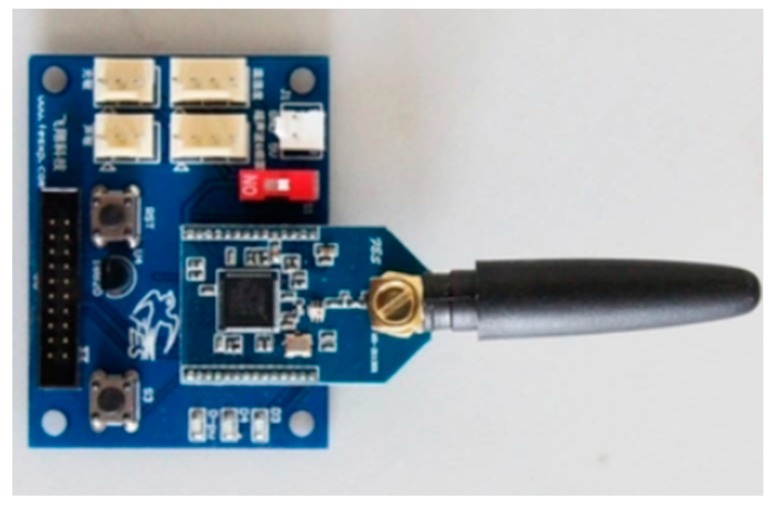
Sensor network node physical map.

The physical map of the nodes in the sensor network is shown in Figure 3.

**Figure 4:**
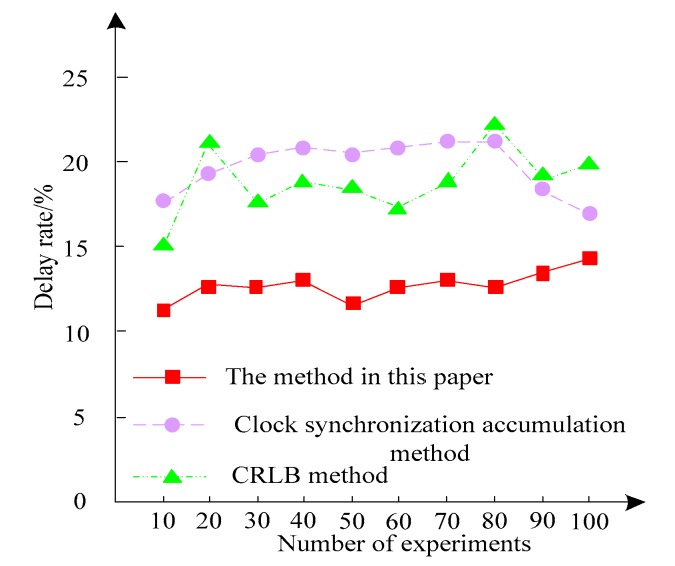
Different methods of sensing communication delay rate.

In order to effectively verify the effectiveness of the proposed method in compensating for the clock error of the sensing network, the method is compared with the clock synchronization accumulation method and the CRLB method. Three methods were used to sense communication, and the communication delays of the three methods were counted in 10 experiments. The comparison results are shown in Figure 4.

It can be seen from Figure 4 that in 100 experiments, the sensing communication delay rate is between 10% and 15% using the method of the present invention. The CRLB method is used to sense the communication delay rate up to 22%, and the clock synchronization accumulation method is used to sense the communication delay rate as high as 21%. The sensing communication delay rate using this method is significantly lower than the CRLB method and the clock synchronization accumulation method. This shows that using the method of this paper to compensate the compensation network clock error can ensure the communication quality of the sensor network.

**Figure 5:**
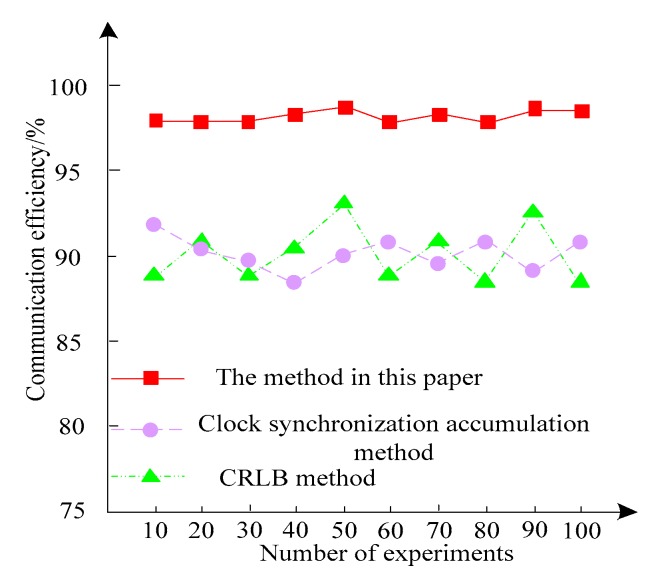
Comparison of communication efficiency between different methods.

Three methods are used to compensate the clock error of the sensor network, and the communication efficiency of the three methods in the 10 experiments is statistically analyzed. The comparison results are shown in Figure 5.

It can be seen from Figure 5 that the clock error communication efficiency of the sensor network is over 97% and the robustness is better. The CRLB method and the clock synchronization accumulation method compensate the clock error communication efficiency of the sensor network by making it less than 93%. The experimental results show that the proposed method compensates the clock error of the sensor network with extremely high communication efficiency.

According to the above experimental results, it can be known that this method is used to compensate the clock error of the sensor network, and the communication delay rate and communication efficiency are better than the CRLB method and the clock synchronization accumulation method. This fully demonstrates the superiority of the method in this paper.

Three methods are used to compensate the clock error of the sensor network, and the statistical simulation time is the information overhead of the three methods of sensing communication within 100 s. The comparison results are shown in Table 2.

It can be seen from the experimental results in Table 2 that the information overhead required to compensate the clock error of the sensor network by this method is significantly smaller than the CRLB method and the clock synchronization accumulation method. Using the method of this paper to compensate, the clock error of the sensor network is only 0.87 J in the experimental that ran for 100 s, and the CRLB method to compensate the clock error of the sensor network is as high as 1.72 J in the experiment that ran for 100 s. 

**Figure 6:**
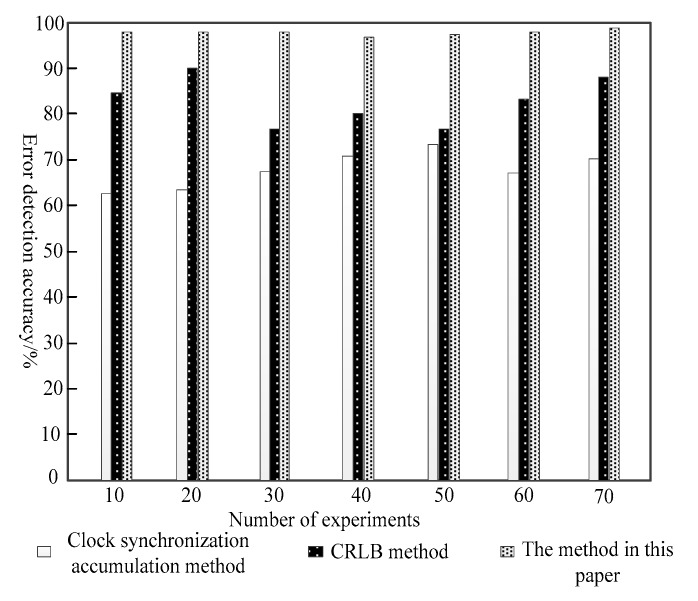
Comparison of error detection accuracy of three methods.

On the basis of the above experiments, it is possible to verify the accuracy of error detection of different methods. The higher the error detection rate, the better the error supplement effect. The experimental results are shown in Figure 6.

Analysis of the experiment results shows that for the clock synchronization accumulation method, the error detection rate is between 63% and 72%, which is the lowest in the three methods. In contrast, for the CRLB method, the error detection rate is between 78% and 89%, and the method of error detection accuracy is over 97%, which was the highest of three methods for error detection precision. Thus, the CRLB method improves the precision of the sensor network error and has laid a solid foundation.

The experimental results again verify the superiority of using this algorithm to compensate the clock error of the sensor network.

**Figure 7:**
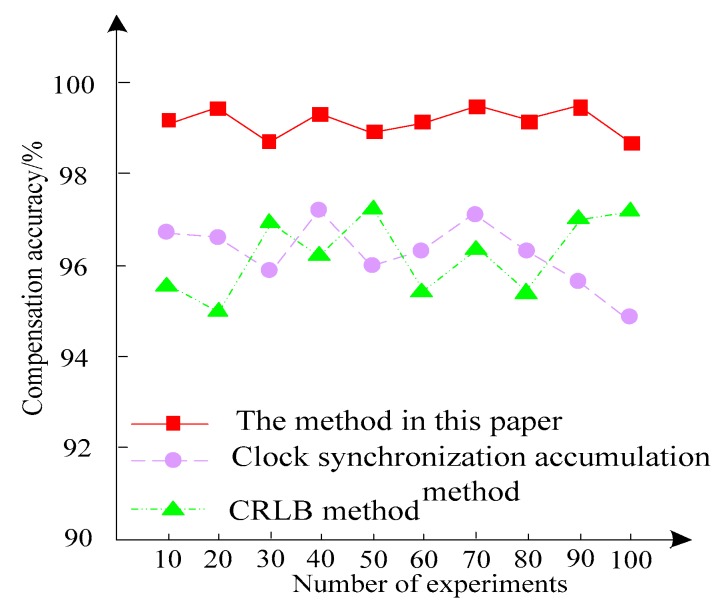
Comparison of compensation accuracy of three methods.

Three methods are used to compensate the clock error of the sensor network, and the compensation accuracy of the three methods of sensor communication is counted in 100 experiments. The comparison results are shown in Figure 7.

It can be seen from the experimental results in Figure 7 that the compensation accuracy of the clock error of the sensor network using this method is above 98%. The compensation accuracy of the clock error of the sensor network using the CRLB method is only 97.7%, and the compensation accuracy of the clock error of the sensor network using the clock synchronization accumulation method is only 97.6%. Through the comparison of the compensation accuracy of the three methods, it can be seen that the compensation of the clock error of the sensor network is higher by using the algorithm, and the high precision can be maintained under different experimental times. This verifies the superiority of the clock error compensation of the proposed algorithm.

In order to verify the effectiveness of the proposed algorithm to compensate the sensor network clock error, the compensation performance of the proposed algorithm is evaluated by the computational complexity of the three methods. The comparison results are shown in Table 3.

It can be seen from Table 3 that when the algorithm is used to compensate the sensor network clock error and the number of nodes is 10, the CPU running time is only 0.17 s. The clock synchronization accumulation method is used to compensate the clock error of the sensor network. When the number of nodes is 10, the CPU running time is as high as 1.31 s. Then, the CRLB method is used to compensate the clock error of the sensor network. When the number of nodes is 10, the CPU running time is as high as 1.71 s. Thus, the CRLB method compensates the clock error of the sensor network. When the number of nodes is 10, the CPU running time is 100 times that of the method, and the algorithm complexity and the number of nodes increase linearly. The experimental results show that the proposed method has low computational complexity and can be applied to large-scale networks of sensor nodes with low cost and low computational energy.

**Figure 8:**
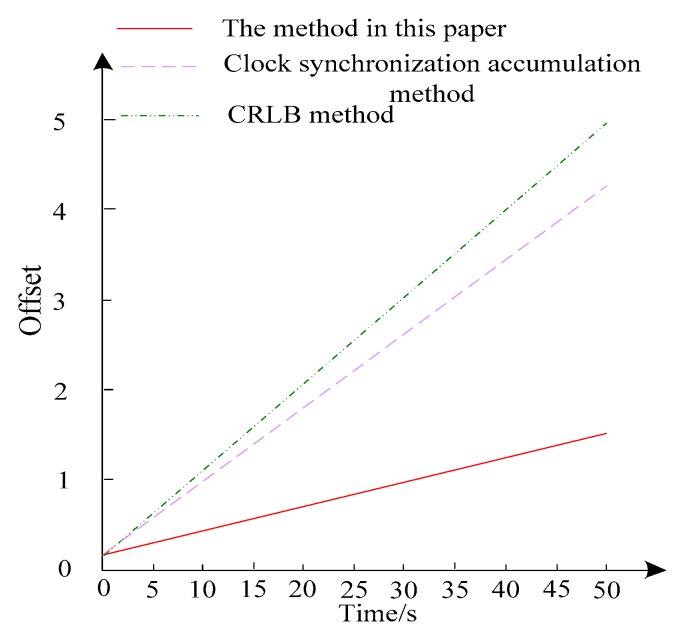
Clock drift diagram of unknown nodes.

In order to visually compare the clock skew and deviation, the clock error of the unknown node compensated by three different methods is compared with the timing of the whole network. The comparison results are shown in Figure 8.

As can be seen from the results of Figure 8, after the synchronization information is transmitted at time 0, the synchronization time exchange is not performed. The method in this paper can effectively suppress the clock drift caused by the increase of time and effectively control the increase of clock skew, effectively reducing the node synchronization frequency. It reduces energy consumption while ensuring compensation accuracy.

## 4. Discussion

The timing mechanism of the nodes in the sensor network consists of a crystal oscillator with a nominal frequency and a count register. Each time an oscillation pulse occurs, the value is incremented by one when counting the calculator. Read the value of the count register to get the current clock value. Ideally, the vibration pulse output of a crystal oscillator of a rated frequency is periodic, and the oscillation period cannot be changed. This oscillation output is called the local physical time. The value in the count register can be read and changed by the local software time. The main causes of inconsistency in the local time of the sensor nodes are:

1. Due to the low-cost characteristics of the node, a quartz crystal oscillator is generally used. The imperfection of the crystal quality results in a pulse frequency that is not absolutely consistent, which makes the node counter count frequency different. The time error caused by this problem is called clock skew, which is usually between 1 and 100 ppm.

2. The node opening time is inconsistent. Due to the hardware manufacturing of the nodes, the turn-on time clocks of the individual sensor nodes are not completely consistent. Especially in large-scale networks, deployed nodes are not all started at the same time. This directly leads to a difference between the initial phases of the nodes. Time inconsistencies due to this cause are often referred to as clock skew.

3. The crystal frequency of the node is greatly affected by the ambient temperature, light intensity, humidity, and electromagnetic interference. At the same time, the voltage and current fluctuations of the node’s own power supply module also have an effect on the frequency variation of the crystal oscillator. In addition, as the node uses time, the aging of the device and other issues will also affect the stability of the crystal oscillator pulse frequency. This cause causes the clock skew to change constantly—that is, the clock drifts.

The main problem to be solved by clock error compensation is to keep the node time consistent by changing the software time and minimize the time error caused by time offset and time drift.

Local clock synchronization issues and wireless link transmission are fundamental quality of service requirements for wireless networks. On the one hand, wireless transmission provides a platform and guarantee the synchronization of the local clock, and the synchronization of the local clock in turn can promote the application development of a series of signal processing and communication platforms.

The compensation of clock error is the main supporting technology of the upper layer coordination mechanism, which determines whether the data is valid in time-sensitive applications. In the time-related observation events, applications such as data fusion, event monitoring, TDOA positioning, and sensing time sequencing all have strict requirements for clock synchronization.

As a non-traditional complex mission-based network, real-time interaction and collaborative processing of information is one of the key QoS guarantees for wireless sensor networks. Due to the lack of resources in a single node, the three pieces of data perceived, processed, and transmitted by the wireless sensor network need to be completed through a specific coordination mechanism. High-precision clock synchronization ensures the most efficient coordination and efficient time-division multiplexing and media access for wireless channels. This improves wireless channel utilization and ensures overall network performance. The clock synchronization performance also determines whether the MAC protocol, dormancy scheduling, and time division multiplexing determine the effective utilization of many resources such as energy and broadband, which further determines the efficiency of energy supply of each node and the life cycle of the entire network.

In the mobile target tracking problem in the context of wireless sensor networks, the node detects the target. Then, the related physical information and the time label are merged into a data packet, and the neighbor nodes that monitor the target communicate with each other at a certain frequency. The purpose is to exchange data packets and obtain motion state information such as the position and speed of the target at a specific moment. This process continues to circulate, with the ultimate goal of obtaining the trajectory of the target within the detection area. Each point on the motion trajectory represents the motion state of the target at a certain moment, and the timing of the time determines the direction of the trajectory. Therefore, clock synchronization plays a key role at both the application and network layers. In the application layer, the purpose of clock synchronization between nodes is to provide accurate protection and processing of data, such as target location and event detection. At the network layer, clock synchronization provides the basis for real-time data transmission, such as node wake-up and packet scheduling. Therefore, the clock synchronization mechanism can penetrate every data-related mitigation, and its implementation directly determines the merits of the data-centric wireless sensor network system.

In order to solve the problem of clock error compensation in sensor networks, a clock error compensation method based on cyclic symmetry algorithm is proposed. The effectiveness of the proposed method is verified by simulation experiments. Simulation results show that the communication delay rate of the proposed method is between 10% and 15%, and the communication delay is low. The clock error communication efficiency is above 97% and the robustness is good. At 100 s, the experiment was only 0.87 J, with high efficiency. The accuracy of clock error detection in sensor networks is always above 97%. The compensation accuracy of clock error is above 98% and there is low computational complexity; thus, the proposed method can effectively suppress clock drift caused by time increase. On the whole, compared with the existing methods, this method has more obvious advantages. The reason why this method has the advantages above is that it constructs the cyclic symmetry matrix of the sensor network based on the basic theory of cyclic symmetry. In the communication process, all nodes are extended to obtain the accumulative delay rate of the sensor network in the specified time domain. By using the accumulated delay rate of the loop network and sensor network, an auto-regressive integral sliding mode control model is established to compensate the accumulated error of clock synchronization, so it has certain practical application value in practice.

At present, many results have been achieved for the time synchronization technology of wireless sensor networks, but there are still many specific problems to be further studied.

At present, the work mainly focuses on the synchronization accuracy with the node, and lacks thinking and research on the synchronization performance of the entire network, which leads to a very high resynchronization frequency and a large communication energy consumption. However, the theory has shown that most of the energy of the sensor node is consumed in the wireless communication module.

Existing synchronization algorithms mostly use maximum likelihood estimation to estimate clock skew and clock skew. This algorithm must use a search algorithm under the nonlinear clock model, which results in very high complexity, does not necessarily have a maximum value, and is not suitable for local calculation of sensor network nodes. This algorithm estimates the clock skew and clock skew of the node in the linear clock model only for short-term applications such as target location.

The synchronization protocol does not consider the relationship between environmental factors and hardware drift from the underlying physical level, especially in low-duty cycle networks. When the node is sleeping, it cannot communicate and transmit, which makes it impossible to synchronize time, and it is impossible to achieve “consistent collective recovery” after a long period of sleep.

In view of the problems in the research of sensor network time synchronization technology mentioned above, the main work of this paper is as follows: Using cyclic symmetry theory to provide an effective solution for the clock synchronization of wireless sensor networks, especially sleep sensor networks, so that node communication energy consumption is greatly reduced. The node can compensate the clock deviation in software mode in the sleep state, and the effectiveness of the algorithm is verified by experiments.

There are still a lot of questions about wireless sensor networks that deserve further study:

1. Delay distribution algorithm

At present, the distribution models of non-deterministic network delays commonly used include Gaussian, exponential, gamma, and Weibull models. However, in a particular wireless sensor network, it is difficult to assess which distribution model of the network’s delay matches the overlay function of those models. Since various factors can affect the distribution of network delays, simulations show that existing algorithms are usually valid for models with a specific delay profile.

2. Security issues with clock synchronization

The security issue of clock synchronization in wireless sensor networks is a relatively new area of research. Most of the existing literature focuses on a single attack mode. Assuming that the attack nodes are sparsely distributed in the network, consider the security issue of clock synchronization. There are still many problems worth considering for the clock synchronization security when considering different attack modes and scenarios in which the attack nodes are concentrated.

3. Large-scale network synchronization

At present, the optimal estimation method of the optimal clock for large-scale wireless sensor network synchronization is also a very important and challenging subject. In this respect, graph theory algorithms are combined with distributed or sequential Bayesian methods, MCMC techniques, and distributed nonlinear optimization techniques. Perhaps it can provide a solution for developing efficient synchronization algorithms in large-scale wireless sensor networks.

4. Joint estimation of synchronization and positioning

In practice, the location of the beacon node is usually given by GPS, and the clock skew and clock skew of the beacon node are also estimated during the sensor network synchronization process. Therefore, in the case of error in the beacon node information, optimizing the estimation problem of the unknown node is also a problem that needs to be solved at present.

## 5. Conclusions

The compensation of the clock error in the sensor network can provide a standard time for the wireless sensor network to refer to, which provides the basic premise for real-time interaction and the collaborative processing of sensor node information and the scheduling of network time. The compensation of clock error is one of the most basic and important problems in wireless sensor networks, and it has always been a hot issue in wireless sensor networks. In this paper, based on the theory of cyclic symmetry, an autoregressive integral sliding control model is established to compensate the cumulative error of clock synchronization in the sensor network. The experimental results show that using this method to compensate the clock error of the sensor network can greatly reduce the communication delay rate of the sensor network and improve its communication efficiency.

## Figures and Tables

**Table 1:** Experimental environment settings.

Project	Parameter
CPU	Intel Xeon
CPU hard disk capacity	1.5 T
CPU frequency	1.2 GHz
Random access memory	128 GB
Operating system	Windows 10
Monitor resolution	1280*1024
Interface type	USB
Simulation software	Matlab 7.0

**Table 2:** Comparison of information overhead of different methods.

Simulation Time/s	CRLB Method/J	Clock Aynchronization Accumulation Method/J	The Method in This Paper/J
0	2	2	2
10	1.99	1.98	1.97
20	1.97	1.92	1.89
30	1.95	1.88	1.81
40	1.92	1.83	1.73
50	1.89	1.72	1.61
60	1.85	1.63	1.52
70	1.81	1.58	1.41
80	1.78	1.52	1.32
90	1.75	1.41	1.21
100	1.72	1.35	0.87

**Table 3:** CPU runtime comparison.

Number of Nodes/N	The Method in This Paper/s	Clock Synchronization Accumulation Method/s	CRLB Method/s
1	0.03	0.78	0.67
2	0.06	0.79	0.85
3	0.07	0.82	1.05
4	0.08	0.89	1.15
5	0.09	0.91	1.27
6	0.11	0.95	1.37
7	0.12	0.97	1.41
8	0.13	1.15	1.57
9	0.15	1.13	1.63
10	0.17	1.31	1.71
